# Prevalence, antimicrobial resistance, virulence gene profile and molecular typing of *Campylobacter* species isolated from poultry meat samples

**DOI:** 10.1002/vms3.944

**Published:** 2022-10-17

**Authors:** Maryam Hadiyan, Hassan Momtaz, Amir Shakerian

**Affiliations:** ^1^ Department of Microbiology, Shahrekord Branch Islamic Azad University Shahrekord Iran; ^2^ Faculty of Veterinary Medicine, Department of Food Hygiene, Shahrekord Branch Islamic Azad University Shahrekord Iran

**Keywords:** antimicrobial resistance, *Campylobacter coli*, *Campylobacter jejuni*, molecular typing, raw poultry meat, virulence factors

## Abstract

**Background:**

*Campylobacter jejuni* and *Campylobacter coli* are the most significant *Campylobacter* species responsible for severe gastrointestinal disorders. Raw poultry meat is considered a source of *Campylobacter* transmission to the human population.

**Objectives:**

The present study was aimed to assess the prevalence rate, antibiotic resistance properties, virulence characters and molecular typing of *C. jejuni* and *C. coli* strains isolated from raw poultry meat samples.

**Methods:**

Three hundred and eighty raw poultry meat samples were collected and analysed for the presence of *Campylobacter* spp. using the microbial culture. Species identification was done using the Polymerase Chain Reaction. Disk diffusion was developed to assess the antimicrobial resistance pattern of isolates. The distribution of virulence and antimicrobial resistance genes was determined by PCR. Enterobacterial Repetitive Intergenic Consensus‐PCR was used for molecular typing.

**Results:**

*Campylobacter* species were isolated from 6.25% of examined samples. *C. jejuni* and *C. coli* contamination rates were found to be 57.44% and 48.14%, respectively. *C. jejuni* strains harboured the highest resistance rate against serythromycin (42.59%), ampicillin (38.88%), ciprofloxacin (33.33%), chloramphenicol (31.48%) and tetracycline (31.48%). *C. coli* isolates harboured the highest resistance rate against ampicillin (73.07%), ciprofloxacin (73.07%), erythromycin (65.38%) and chloramphenicol (50%). *AadE1* (44.44%), *blaOXA‐61* (42.59%) and *tet(O)* (35.18%) were the most commonly detected resistance genes in *C. jejuni* and *cmeB* (34.61%) and *blaOXA‐61* (34.61%) were the most commonly detected among *C. coli* strains. The most frequent virulence factors among the *C. jejuni* isolates were *flaA* (100%), *ciaB* (100%), *racR* (83.33%), *dnaJ* (81.48%), *cdtB* (81.48%), *cdtC* (79.62%) and *cadF* (74.07%). The most frequent virulence factors among the *C. coli* isolates were *flaA* (100%), *ciaB* (100%), *pldA* (65.38%) and *cadF* (61.53%).

**Conclusions:**

The majority of *C. jejuni* and *C. coli* strains had more than 80% similarities in their ERIC‐PCR pattern, which may show their common source of transmission. The role of goose and quebec meat samples as reservoirs of virulent and antimicrobial resistant *Campylobacter* spp. was determined.

## Graphical abstract

Chicken and Turkey meat samples harboured the highest contamination rates. *C. jejuni* and *C. coli* isolates harboured a high resistance rate against some antibiotics, which was accompanied by the high distribution of most antimicrobial resistance‐encoding genes.

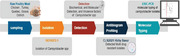

## 1 INTRODUCTION

*Campylobacter* species (spp.) are important foodborne pathogens responsible for the majority of cases of enteric infections known as campylobacteriosis in developed and developing countries (Abukhattab et al., 2022). In recent years, about 500 million cases of gastrointestinal infections due to the *Campylobacter* species have been reported globally (Marotta et al., 2019). In 2017, campylobacteriosis is determined as the most common zoonotic disease with about 246,000 confirmed cases and a morbidity rate of 64.8 per 100,000 population in the European Union [European Food Safety Authority and European Centre for Disease Prevention and Control (EFSA and ECDC), 2017].

*Campylobacter jejuni* (*C. jejuni*) and *C. coli* are major species responsible for severe cases of human gastroenteritis (Igwaran & Okoh, 2019). Humans most often become infected by ingesting contaminated food, particularly undercooked poultry meat (Myintzaw et al., 2022). Poultry carcasses are typically contaminated during defeathering and evisceration by faeces leakage containing campylobacters from the cloaca (Hakeem & Lu, 2021). In most cases, campylobacteriosis is typically self‐limiting; however, complications may occur in some persons. Around 1 in 1000 infected individuals develops Guillain–Barré syndrome (GBS), a thoughtful autoimmune‐mediated neurological disorder that causes weakness of extremities, complete paralysis, respiratory insufficiency and death (Scallan Walter et al., 2020).

Diseases caused by *Campylobacter* spp. are commonly occurred due to the presence and activity of diverse kinds of virulence factors. In this regard, cytolethal distending toxin (*cdt*), phospholipase A outer membrane (*pldA*), IV secretory system (*virB11*), flagellar gene (*flaA*), *Campylobacter* invasion antigen B (*ciaB*), *Campylobacter* adhesion to fibronectin (*cadF*), regulatory protein R (*racR*), chaperone protein (*dnaJ*), Guillain‐Barré syndrome associated genes (*cgtB* and *wlaN*), and enterochelin binding lipoprotein encoded by siderophore transport (*ceuE*) are responsible for the adhesion and invasion of *Campylobacter* spp. to the human epithelial cells (Hassan et al., 2019).

Recent reports revealed the high resistance rate of *Campylobacter* spp. strains towards different types of antimicrobial agents (Audu et al., 2022). Antimicrobial‐resistant *Campylobacter* strains caused more severe infections for a longer time with a higher economic burden (Luangtongkum et al., 2009). *Campylobacter* spp. strains isolated from human clinical infections and poultry sources harboured high resistance towards aminoglycosides, tetracyclines, penicillins, quinolones, cephalosporins, phenicols, macrolides and β‐lactams antimicrobials (Hlashwayo et al., 2020; Yang et al., 2019). In this regard, kanamycin‐resistance determinant (*aphA‐3*), multidrug efflux pump gene (*cmeB*), tetracyclines resistance encoding gene (*tet(O*)), β‐lactams resistance gene (*blaOXA‐61*) and aminoglycosides determinant gene (*aadE1*) were predominant among the *Campylobacter* strains isolated from resistance cases (Elhadidy et al., 2020; Pérez‐Boto et al., 2014).

Diverse Polymerase Chain Reaction (PCR)‐based typing, such as PCR sequencing, PCR‐ribotyping and enterobacterial repetitive intergenic consensus PCR (ERIC‐PCR) have been developed for the molecular typing of *Campylobacter* spp. (Igwaran & Okoh, 2020a). ERIC‐PCR technique is a simple tool used to differentiate bacteria strains isolated from diverse sources. This technique is a strong tool for the exploration of prokaryotic genomes and has been reported to have improved reproducibility and high discriminatory power (Bilung et al., 2018). Its application for successful typing of *Campylobacter* spp. has been reported in a previous survey (Staji et al., 2018).

Data about the epidemiology of foodborne campylobacteriosis are scarce in Iran. Additionally, the exact prevalence rate, virulence characters and antimicrobial resistance of *Campylobacter* spp. were not well defined among poultry in Iran. Thus, the present survey was done to assess the prevalence rate, antimicrobial resistance pattern, distribution of virulence genes and the molecular typing of *C. coli* and *C. jejuni* strains isolated from poultry meat samples.

## 2 MATERIALS AND METHODS

### 2.1 Samples

A total of 380 raw poultry meat samples, including chicken (*n* = 120), turkey (*n* = 55), quebec (*n* = 65), goose (*n* = 65) and ostrich (*n* = 75) were randomly collected from retail poultry meat centres, Shahrekord, Iran. Raw poultry meat samples (100 g) were collected from the thigh muscle using sterile plastic bags. Samples were transferred in refrigerated containers at 4°C. Samples transportation and processing were done within 2 h after collection.

### 2.2 Bacterial isolation and identification

*Campylobacter* spp. isolation was done according to the EN ISO 10272–1:2006 method (ISO 10272‐1, 2006). Twenty‐five grams of meat were inoculated into 225 ml of Bolton broth (Oxoid) containing the Bolton broth selective supplement (Oxoid) and 5% laked horse blood (Oxoid). Following, bacterial suspension was spread onto Charcoal Cefoperazone Deoxycholate Agar (CCDA) (Oxoid, Basingstoke, United Kingdom) plates, and then incubated for 48 h at 42°C under microaerobic conditions (85% N_2_, 5% O_2_ and 10% CO_2_). A colony from each medium was subjected to biochemical examinations, including Gram‐staining, catalase production (3% H2O2), hippurate oxidase and hydrolysis, indoxyl acetate hydrolysis, urease activity and resistance against cephalothin (Nachamkin, 2003). *Campylobacter* species identification was done using the PCR (Denis et al., 1999). Suspected *Campylobacter* isolates were sub‐cultured on Bolton broth and incubated for 48 h at 42°C in a microaerobic condition. According to the manufacturer's instructions, the genomic DNA was extracted from the isolates using the DNA extraction kit (Thermo Fisher Scientific, St. Leon‐Rot, Germany). The purity (A260/A280) and concentration of the extracted DNA were then checked (NanoDrop, Thermo Scientific, Waltham, MA, USA). Furthermore, the DNA's quality was assessed on a 2% agarose gel stained with ethidium bromide (0.5 μg/ml) (Thermo Fisher Scientific, St. Leon‐Rot, Germany). The first primers set was used for detection of *Campylobacter* genus *16S rRNA* gene (F: 5′‐ATCTAATGGCTTAACCATTAAAC‐3′ and R: 5′‐GGACGGTAACTAGTTTAGTATT‐3′) (857 bp). The second one was used to detect *C. jejuni mapA* gene (F: 5′‐CTATTTTATTTTTGAGTGCTTGTG‐3′ and R: 5′‐GCTTTATTTGCCATTTGTTTTATTA‐3′) (589 bp). The third one was used to detect *C. coli ceuE* gene (F: 5′‐AATTGAAAATTGCTCCAACTATG‐3′ and R: 5′‐TGATTTTATTATTTGTAGCAGCG‐3′) (462 bp) (Rahimi et al., 2010).

### 2.3 Antimicrobial resistance pattern

To investigate the pattern of antimicrobial resistance of *C. jejuni* and *C. coli* isolates, the simple disk diffusion method (Kirby Baeur) was used. The bacteria were incubated on Mueller‐Hinton agar (Merck, Germany) containing 5% (vol/vol) sheep blood at 42°C under a microaerophilic atmosphere in the presence of diverse antimicrobial discs, including gentamicin (10 μg/disk), ciprofloxacin (5 μg/disk), nalidixic acid (30 μg/disk), tetracycline (30 μg/disk), ampicillin (10 μg/disk), amoxicillin (30 μg/disk), erythromycin (15 μg/disk), azithromycin (15 μg/disk), clindamycin (2 μg/disk) and chloramphenicol (30 μg/disk). The interpretation was done using the guidelines of the Clinical and Laboratory Standard Institute (CLSI, 2021). *C. jejuni* ATCC 33560 and *C. coli* ATCC 33559 were used as controls in antimicrobial susceptibility testing.

### 2.4 Detection of virulence factors and antimicrobial resistance‐encoding genes

Table 1 shows the PCR conditions met to detect antimicrobial resistance genes and virulence factors (Datta et al., 2003; Obeng et al., 2012). A programmable DNA thermocycler (Eppendorf Mastercycler 5330, Eppendorf‐Nethel‐Hinz GmbH, Hamburg, Germany) was used in all PCR reactions. In addition, amplified samples were analysed by electrophoresis (120 V/208 mA) in a 2.5% agarose gel stained with 0.1% ethidium bromide (0.4 μg/ml). Besides, UVI doc gel documentation systems (Grade GB004, Jencons PLC, London, UK) were used to analyse images.

**TABLE 1:** Primers of virulence and antibiotic resistance genes, annealing temperatures and size of amplicons

Gene	Primer sequences (5′–3′)	Annealing temperatures (°C)	Product size (bp)
*23S rRNA*	TTAGCTAATGTTGCCCGTACCG TAGTAAAGGTCCACGGGGTCGC	46	485
*recR*	GATGATCCTGACTTTG TCTCCTATTTTTACCC	45	584
*dnaJ*	AAGGCTTTGGCTCATC CTTTTTGTTCATCGTT	46	720
*wlaN*	TTAAGAGCAAGATATGAAGGTG CCATTTGAATTGATATTTTTG	46	672
*Virbll*	TCTTGTGAGTTGCCTTACCCCTTTT CCTGCGTGTCCTGTGTTATTTACCC	53	494
*cdtC*	CGATGAGTTAAAACAAAAAGATA TTGGCATTATAGAAAATACAGTT	47	182
*cdtB*	CAGAAAGCAAATGGAGTGTT AGCTAAAAGCGGTGGAGTAT	51	620
*cdtA*	CCTTGTGATGCAAGCAATC ACACTCCATTTGCTTTCTG	49	370
*flaA*	AATAAAAATGCTGATAAAACAGGTG TACCGAACCAATGTCTGCTCTGATT	53	585
*cadF*	TTGAAGGTAATTTAGATATG CTAATACCTAAAGTTGAAAC	45	400
*pldA*	AAGCTTATGCGTTTTT TATAAGGCTTTCTCCA	45	913
*ciaB*	TTTTTATCAGTCCTTA TTTCGGTATCATTAGC	42	986
*ceuE*	CCTGCTACGGTGAAAGTTTTGC GATCTTTTTGTTTTGTGCTGC	48.9	793
*cgtB*	TAAGAGCAAGATATGAAGGTG GCACATAGAGAACGCTACAA	49.9	561
*tet*(O)	GCGTTTTGTTTATGTGCG ATGGACAACCCGACAGAAG	54	559
*cme*B	TCCTAGCAGCACAATATG AGCTTCGATAGCTGCATC	54	241
*bla*_OXA‐61_	AGAGTATAATACAAGCG TAGTGAGTTGTCAAGCC	54	372
*aphA‐3‐1*	TGCGTAAAAGATACGGAAG CAATCAGGCTTGATCCCC	54	701

### 2.5 ERIC‐PCR molecular typing

*C. jejuni* and *C. coli* isolates of different raw poultry meat samples were subjected to PCR using the ERIC primer set R1: ATGAAGCTCCTGGGGATTCAC and R2: AAGTAAGTGACTGGGGTGAGCG (Zorman et al., 2006). The PCR reactions were verified by resolving them in 3% agarose gel in a 5× TBE buffer, stained with ethidium bromide at 90 volts for 240 min and viewed. ERIC‐PCR DNA fingerprints were analysed with computer‐assisted pattern analysis using the GelJ v.2.0. software (Heras et al., 2015). The relatedness of the isolates was compared and dendrograms were constructed by UPGMA and cluster analysis was used to determine the relationships between each isolate. The value of discriminatory power [D) was determined using an online calculator for discriminatory power as reported (Milton et al., 2015).

### 2.6 Data assessment

Data analysis was performed by SPSS Statistics 21.0 (SPSS Inc., Chicago, IL, USA). Chi‐square and Fisher's exact two‐tailed tests were performed to assess any significant relationship between the *Campylobacter* prevalence and virulence and antimicrobial resistance properties. Besides, *p* value < 0.05 was considered statistically significant.

## 3 RESULTS

### 3.1 *Campylobacter* distribution

Table 2 shows the *Campylobacter* distribution among the examined samples. Ninety‐four out of 380 (6.25%) raw poultry meat samples were contaminated with *Campylobacter* species. Raw chicken meat samples (61.66%) harboured the highest contamination rate, while raw goose meat (1.53%) harboured the lowest. The total prevalence of *C. jejuni* and *C. coli* among the isolated bacteria were 57.44% and 48.14%, respectively. Fourteen (25.92%) isolates were contaminated with other *Campylobacter* spp. Raw quebec and goose meat samples harboured the highest contamination rate of *C. jejuni* (100% each), while raw ostrich meat samples (50%) harboured the lowest. There were no positive results for *C. coli* contamination in raw quebec and goose meat samples. However, raw turkey meat samples (71.42%) harboured the highest contamination rate of *C. coli*, while raw chicken meat samples (45.23%) harboured the lowest. From a statistical seeing, significant differences were found between types of samples and *Campylobacter* prevalence (*p* < 0.05). Additionally, a significant difference was obtained between the prevalence of *C. jejuni* and *C. coli* (*p* < 0.05).

**TABLE 2:** Campylobacter distribution among the examined samples

Raw meat samples	No. of samples collected	No. (%) of *Campylobacter*‐positive samples	No. (%) of *C. jejuni*‐positive samples	No. (%) of *C. coli*‐positive samples	No. (%) of other *Campylobacter* spp.‐positive samples
Chicken	120	74 (61.66)	42 (56.75)	19 (45.23)	13 (30.95)
Turkey	55	13 (23.63)	7 (53.84)	5 (71.42)	1 (14.28)
quebec	65	2 (3.07)	2 (100.00)	‐	‐
Goose	65	1 (1.53)	1 (100.00)	‐	‐
Ostrich	75	4 (5.33)	2 (50.00)	2 (50.00)	‐
Total	380	94 (6.25)	54 (57.44)	26 (48.14)	14 (25.92)

### 3.2 *Campylobacter* antimicrobial resistance

Table 3 shows the antimicrobial resistance of *C. jejuni* isolates of examined samples. *C. jejuni* strains harboured the highest antimicrobial resistance rate against erythromycin (42.59%), ampicillin (38.88%), ciprofloxacin (33.33%), chloramphenicol (31.48%) and tetracycline (31.48%). The lowest resistance rate was seen towards gentamicin (1.85%) and amoxicillin (14.81%). *C. coli* isolates harboured the highest antimicrobial resistance rate against ampicillin (73.07%), ciprofloxacin (73.07%), erythromycin (65.38%) and chloramphenicol (50%). The lowest resistance rate was seen for amoxicillin (23.07%), azithromycin (30.76%) and tetracycline (34.61%). No resistance was found towards gentamicin. From a statistical seeing, significant differences were found between types of samples and *Campylobacter* antimicrobial resistance rate (*p* < 0.05).

**TABLE 3:** Antimicrobial resistance of *C. jejuni* isolates of examined samples

	No. (%) of *C. jejuni* isolates harboured resistance against each antimicrobial agent									
Samples (No. of *C. jejuni* positive)	*GM10* *	*CIP5*	*NA30*	*TE30*	*AM10*	*AMC30*	*E15*	*AZM15*	*CC2*	*C30*
Chicken (42)	‐	13 (30.95)	7 (16.66)	12 (28.57)	15 (35.71)	7 (16.66)	18 (42.85)	9 (21.42)	9 (21.42)	12 (28.57)
Turkey (7)	1 (14.28)	2 (28.57)	3 (42.85)	2 (28.57)	3 (42.85)	‐	2 (28.57)	1 (14.28)	2 (28.57)	3 (42.85)
quebec (2)	‐	2 (100)	2 (100)	2 (100)	2 (100)	‐	2 (100)	1 (50)	1 (50)	1 (50)
Goose (1)	‐	‐	‐	1 (100)	1 (100)	‐	1 (100)	‐	1 (100)	‐
Ostrich (2)	‐	1 (50)	‐	‐	‐	1 (50)	‐	‐	‐	1 (50)
Total (54)	1 (1.85)	18 (33.33)	12 (22.22)	17 (31.48)	21 (38.88)	8 (14.81)	23 (42.59)	11 (20.37)	13 (24.07)	17 (31.48)

	**No. (%) of *C. coli* isolates harboured each virulence factor**									
Samples (No. of *C. coli* positive)	***GM10***	***CIP5***	***NA30***	***TE30***	***AM10***	***AMC30***	***E15***	***AZM15***	***CC2***	***C30***
Chicken (19)	‐	13 (68.42)	7 (36.84)	7 (36.84)	13 (68.42)	6 (31.57)	15 (78.94)	8 (42.10)	9 (47.36)	8 (42.10)
Turkey (5)	‐	4 (80)	3 (60)	1 (20)	4 (80)	‐	1 (20)	‐	2 (40)	4 (80)
Ostrich (2)	‐	2 (100)	2 (100)	1 (50)	2 (100)	‐	1 (50)	‐	1 (50)	1 (50)
Total (26)	‐	19 (73.07)	12 (46.15)	9 (34.61)	19 (73.07)	6 (23.07)	17 (65.38)	8 (30.76)	12 (46.15)	13 (50)

* G10: gentamicin (10 μg/disk), CIP5: ciprofloxacin (5 μg/disk), NA30: nalidixic acid (30 μg/disk), TE30: tetracycline (30 μg/disk), AM10: ampicillin (10 μg/disk), AMC30: amoxicillin (30 μg/disk), E15: erythromycin (15 μg/disk), AZM15: azithromycin (15 μg/disk), CC2: clindamycin (2 μg/disk), C30: chloramphenicol (30 μg/disk).

### 3.3 Distribution of antimicrobial resistance‐encoding genes

Table 4 shows the antimicrobial resistance‐encoding genes distribution among the *C. jejuni* isolates of examined samples. The most commonly detected antimicrobial resistance‐encoding genes in the *C. jejuni* strains were *aadE1* (44.44%), *blaOXA‐61* (42.59%) and *tet(O)* (35.18%). Among the *C. coli* strains, *cmeB* (34.61%) and *blaOXA‐61* (34.61%) were the most commonly detected antimicrobial resistance‐encoding genes. From a statistical seeing, significant differences were found between types of samples and distribution of antimicrobial resistance‐encoding genes (*p* < 0.05). *C. jejuni* strains harboured a higher and more diverse distribution of antimicrobial resistance‐encoding genes than *C. coli* isolates (*p* < 0.05).

**TABLE 4:** Antimicrobial resistance‐encoding genes distribution among the *C. jejuni* isolates of examined samples

	No. (%) of *C. jejuni* isolates harboured each gene				
Samples (No. of *C. jejuni* positive)	*aphA‐3‐1*	*cmeB*	*tet(O)*	*blaOXA‐61*	*aadE1*
Chicken (42)	1 (2.38)	10 (23.80)	14 (33.33)	17 (40.47)	18 (42.85)
Turkey (7)	1 (14.28)	2 (28.57)	2 (28.57)	3 (42.85)	3 (42.85)
quebec (2)	‐	2 (100)	2 (100)	2 (100)	2 (100)
Goose (1)	‐	‐	1 (100)	1 (100)	1 (100)
Ostrich (2)	‐	1 (50)	‐	‐	‐
Total (54)	2 (3.70)	15 (27.77)	19 (35.18)	23 (42.59)	24 (44.44)

	**No. (%) of *C. coli* isolates harboured each gene**				
Samples (No. of *C. coli* positive)	***aphA‐3‐1***	***cmeB***	***tet(O)***	***blaOXA‐61***	***aadE1***
Chicken (19)	‐	13 (68.42)	9 (47.36)	13 (68.42)	14 (73.68)
Turkey (5)	‐	4 (80)	1 (20)	4 (80)	3 (60)
Ostrich (2)	‐	2 (100)	1 (50)	2 ()100	1 (50)
Total (26)	‐	19 (34.61)	11 (42.30)	19 (34.61)	18 (69.23)

### 3.4 *Campylobacter* virulence characters

Table 5 shows the virulence factors distribution among the *C. jejuni* isolates of examined samples. The most frequent virulence factors among the *C. jejuni* isolates were *flaA* (100%), *ciaB* (100%), *racR* (83.33%), *dnaJ* (81.48%), *cdtB* (81.48%), *cdtC* (79.62%) and *cadF* (74.07%). The lowest distribution rate was related to *wlaN* (9.25%), *virbll* (9.25%) and *cgtB* (24.07%) virulence factors. Additionally, the most frequent virulence factors among the *C. coli* isolates were *flaA* (100%), *ciaB* (100%), *pldA* (65.38%) and *cadF* (61.53%). The lowest distribution rate was related to *cdtA* (11.53%), *cdtB* (19.23%), *cdtC* (19.23%) and *racR* (19.23%) virulence factors. There were no positive results for the *dnaJ*, *wlaN*, *virbll* and *ceuE* virulence factors. From a statistical seeing, significant differences were found between types of samples and distribution of virulence factors (*p* < 0.05). *C. jejuni* strains harboured a higher and more diverse distribution of virulence factors than *C. coli* isolates (*p* < 0.05).

**TABLE 5:** Virulence factors distribution among the *C. jejuni* and *C. coli* isolates of examined samples

	No. (%) of *C. jejuni* isolates harboured each virulence factor												
Samples (No. of *C. jejuni* positive)	*racR*	*dnaJ*	*wlaN*	*virbll*	*cdtC*	*cdtB*	*cdtA*	*flaA*	*cadF*	*pldA*	*ciaB*	*ceuE*	*cgtB*
Chicken (42)	35 (83.33)	35 (83.33)	4 (9.52)	4 (9.52)	35 (83.33)	35 (83.33)	29 (69.04)	42 (100)	31 (73.80)	24 (57.14)	42 (100)	16 (38.09)	10 (23.80)
Turkey (7)	5 (71.42)	4 (57.14)	‐	‐	3 (42.85)	4 (57.14)	4 (57.14)	7 (100)	5 (71.42)	5 (71.42)	7 (100)	3 (42.85)	1 (14.28)
quebec (2)	2 (100)	2 (100)	‐	‐	2 (100)	2 (100)	1 (50)	2 (100)	2 (100)	1 (50)	2 (100)	1 (50)	1 (50)
Goose (1)	1 (100)	1 (100)	1 (100)	1 (100)	1 (100)	1 (100)	1 (100)	1 (100)	‐	1 (100)	1 (100)	1 (100)	1 (100)
Ostrich (2)	2 (100)	2 (100)	‐	‐	2 (100)	2 (100)	2 (100)	2 (100)	2 (100)	‐	2 (100)	‐	‐
Total (54)	45 (83.33)	44 (81.48)	5 (9.25)	5 (9.25)	43 (79.62)	44 (81.48)	37 (68.51)	54 (100)	40 (74.07)	31 (57.40)	54 (100)	21 (38.88)	13 (24.07)

	**No. (%) of *C. coli* isolates harboured each virulence factor**												
Samples (No. of *C. coli* positive)	***racR***	***dnaJ***	***wlaN***	***virbll***	***cdtC***	***cdtB***	***cdtA***	***flaA***	***cadF***	***pldA***	***ciaB***	***ceuE***	***cgtB***
Chicken (19)	3 (15.78)	‐	‐	‐	3 (15.78)	3 (15.78)	3 (15.78)	19 (100)	12 (63.15)	12 (63.15)	19 (100)	‐	8 (42.10)
Turkey (5)	2 (40)	‐	‐	‐	1 (20)	1 (20)	‐	5 (100)	3 (60)	3 (60)	5 (100)	‐	2 (40)
Ostrich (2)	‐	‐	‐	‐	1 (50)	1 (50)	‐	2 (100)	1 (50)	2 (100)	2 (100)	‐	1 (50)
Total (26)	5 (19.23)	‐	‐	‐	5 (19.23)	5 (19.23)	3 (11.53)	26 (100)	16 (61.53)	17 (65.38)	26 (100)	‐	11 (42.30)

### 3.5 ERIC‐PCR molecular typing

**FIGURE 1:**
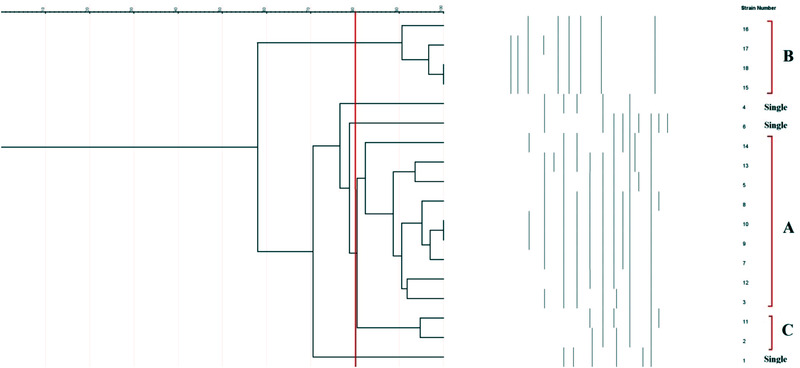
ERIC‐PCR molecular typing of *C. jejuni* isolates of examined samples

Figure 1 shows the ERIC‐PCR molecular typing of *C. jejuni* isolates of examined samples. Rendering a 80% similarity in the genetic bases of *C. jejuni* isolates, bacteria were classified into three different ERIC‐based types. Isolates No. 15 and 18 and also 9 and 10 had a 100% similarity and were classified with each other.

**FIGURE 2:**
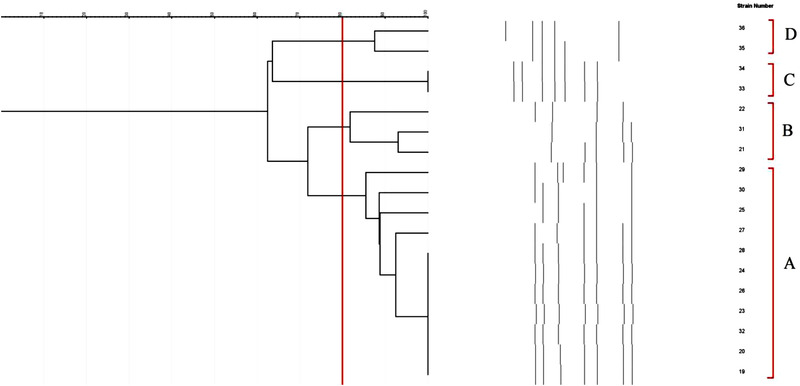
ERIC‐PCR molecular typing of *C. coli* isolates of examined samples

Figure 2 shows the ERIC‐PCR molecular typing of *C. coli* isolates of examined samples. Rendering an 80% similarity in the genetic bases of *C. coli* isolates, bacteria were classified into four different ERIC‐based types. Isolates No. 33 and 34 and also 19, 20, 23, 24, 26, 28 and 32 had a 100% similarity and were classified with each other.

**FIGURE 3:**
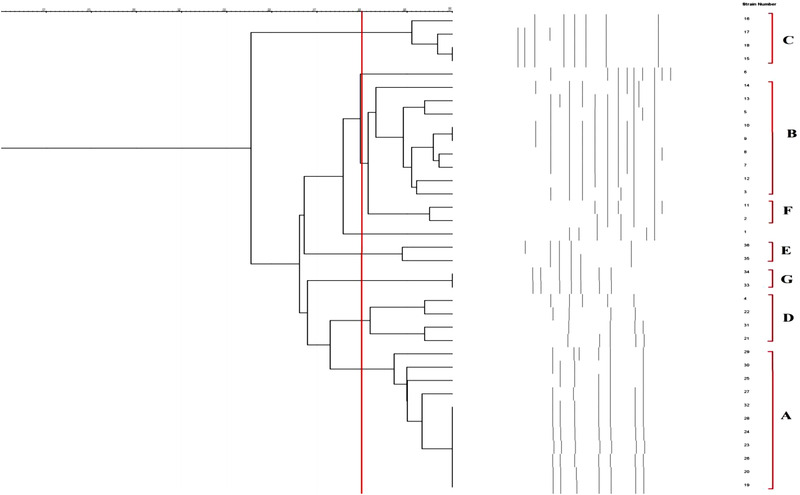
ERIC‐PCR molecular typing of all *Campylobacter* isolates of examined samples

Figure 3 shows the molecular typing of all campylobacter isolates of examined samples. Rendering an 80% similarity in the genetic bases of *C. coli* isolates, bacteria were classified into 7 different ERIC‐based types. Isolates No .15 and 18, 9 and 10, 33 and 34 and finally and also 19, 20, 23, 24, 26, 28 and 32 had a 100% similarity and were classified with each other.

## 4 DISCUSSION

Targeted control of foodborne pathogens usually relies on the identification of sources and routes of transmission. Poultries can harbour *Campylobacter* and represent sources for human campylobacteriosis. All phases – from primary poultry production to the consumer – play an imperative portion in the *Campylobacter* transmission (Guirin et al., 2020).

The present survey was done to assess the prevalence, antimicrobial resistance properties, virulence characters and molecular typing of *C. jejuni* and *C. coli* strains isolated from raw chicken, turkey, ostrich, goose and quebec meat samples. *C. jejuni* and *C. coli* prevalence among the examined samples were 14.21% (54/380) and 6.84% (26/380), respectively. Variation in *Campylobacter* prevalence rate in poultry farms in European countries has been from 0.5% to 13% in Norway, Finland and Sweden and up to 80% in other countries (European Food Safety Authority, 2015). In Australia (Walker et al., 2019), *Campylobacter* spp. was detected in 90% of chicken raw meat and 73% of chicken offal samples. In Iran (Sabzmeydani et al., 2020), the total prevalence of *C. jejuni* and *C. coli* among the raw chicken, turkey, quail, quebec, duck, goose, pheasant and ostrich meat samples were 56.66% and 11.11%, 20% and 22.22%, 42.22% and 2.22%, 26.25% and 1.25%, 37.50% and 5%, 26.66% and 5%, 24% and 2% and finally, 100% and 0%, respectively, which supported our findings of the higher prevalence of *C. jejuni* and *C. coli* in examined samples. A similar survey conducted by Dabiri et al. (2014) reported that the *Campylobacter* prevalence among the chicken meat samples was 44%, in which *C. jejuni* and *C. coli* were identified in 79% and 21% of isolates, respectively. Di Giannatale et al. (2019) showed the prevalence of *Campylobacter* spp. among the poultry meat samples was 17.38% with the higher distribution of *C. jejuni* (58.45% of isolates) than *C. coli* (41.55% of isolates). Szosland‐Fałtyn et al. (2018) reported that the *Campylobacter* spp. the prevalence among raw turkey, chicken, goose and duck meat samples was 18.38%, 49.70%, 6.60% and 43.80%, respectively. They also showed that the prevalence of *C. jejuni* and *C. coli* among the raw chicken, turkey, goose and duck samples were 36.31% and 13.11%, 12.10% and 6.50%, 27.23% and 16.14% and 4.30% and 2.20%, respectively. Probable reasons for differences in the prevalence rate of *Campylobacter* spp. reported in diverse researches are differences in sampling time and location, method of sampling, types of samples, hygienic conditions of poultry farms and even different laboratory techniques. Chicken meat samples harboured the highest contamination rate, which partly may be due to their high number in the slaughter line and the possibility of transferring contamination between the carcasses. Instead, goose and quebec are usually slaughtered in very small numbers and separately from the chickens. As a result, the possibility of transmitting contamination between their carcasses is very low.

*C. jejuni* and *C. coli* isolate harboured a high resistance rate towards examined antimicrobial agents, particularly erythromycin, ampicillin, ciprofloxacin and tetracycline. Unauthorised and improper antimicrobial administration, antimicrobials and disinfectant overuse and self‐medication with antimicrobials can be conceivable reasons for the high prevalence of antimicrobial resistance. Contact of the carcass surface with the slaughterhouse environment and contaminated staff can cause the transfer of antimicrobial‐resistant strains to the poultry carcass surface. High resistance of *Campylobacter* strains towards erythromycin, ampicillin, ciprofloxacin and tetracycline was reported from Ghana (Karikari et al., 2017), South Africa (Igwaran & Okoh, 2020b), Italy (García‐Fernández et al., 2018) and the United States (Noormohamed & Fakhr, 2014). Shakir (2021) reported that resistance rate of *Campylobacter* spp. against erythromycin, tetracycline, ciprofloxacin, chloramphenicol, gentamicin, trimethoprim‐sulphamethoxazole, amoxiclav, ampicillin, ceftriaxone and nalidixic acid antimicrobials were 50%, 88.80%, 100%, 27.70%, 30.50%, 80.50%, 27.70%, 80.50%, 50% and 100%, respectively. In a similar survey, Gharbi et al. (2018) stated that the resistance rate of *C. jejuni* and *C. coli* isolates of poultry meat samples towards ampicillin, amoxicillin/acid clavulanic, ciprofloxacin, nalidixic acid, erythromycin, tetracycline, chloramphenicol and gentamicin antimicrobial agents were 73.60% and 34.10%, 52.70% and 34.10%, 98.90% and 100%, 57.10% and 22%, 100% and 100%, 100% and 100%, 83.50% and 100% and finally 14.30% and 9.80%, respectively, which confirm our findings of the higher antibiotic resistance of *C. jejuni* isolates than that of *C. coli*. However, Giacomelli et al. (2014) reported that the *C. jejuni* strains isolated from poultry harboured more than 50% susceptibility towards gentamicin, apramycin, streptomycin, cefotaxime, amoxicillin + clavulanic acid, erythromycin, tilmicosin, tylosin, clindamycin and chloramphenicol. Reversely, they showed that majority of *C. coli* strains were resistant to streptomycin, cephalothin, cefotaxime, ampicillin, nalidixic acid, ciprofloxacin, erythromycin, tilmicosin, tylosin, tetracycline, clindamycin and chloramphenicol. As a result, Giacomelli et al. (2014) report represents the contradiction in the results obtained in the present study in terms of higher resistance of *C. coli* than *C. jejuni* strains and high susceptibility of *C. jejuni* strains to antimicrobials that were highly resistant in our study. The reason for the difference in the extent and pattern of antimicrobial resistance in different studies is probably the availability or non‐availability of antimicrobials, the presence or absence of strict rules for prescribing antimicrobials and finally the difference in the personal opinion of veterinarians in prescribing antimicrobials. The prevalence of resistance to amoxicillin, azithromycin and clindamycin was relatively lower than that of other antibiotics. Amoxicillin, azithromycin and clindamycin are human‐prescribed antibiotics in the hospital and are not used in veterinary medicine. Thus, it is not surprising that *C. coli* than *C. jejuni* strains harboured a lower resistance rate against them. Another important finding was the high resistance rate of bacteria towards chloramphenicol (31.48% in *C. jejuni* and 50% in *C. coli* strains). Chloramphenicol is an illicit drug with a limited prescription. However, the use of this antibiotic illegally is done only in poultry farms in Iran. Thus, it is not surprising that a high resistance rate against this antimicrobial agent was reported. Similarly, high resistance of *C. jejuni* and *C. coli* strains against chloramphenicol was reported from Kenya (Nguyen et al., 2016), China (Li et al., 2017) and Iran (Fani et al., 2019).

Antimicrobial resistance among the *C. coli* than *C. jejuni* strains was associated with the presence of antimicrobial resistance‐encoding genes, particularly *cmeB*, *tet(O)*, *blaOXA‐61* and *aadE1* genes. Scarce data are available about the distribution of antimicrobial resistance‐encoding genes in *Campylobacter* strains isolated from poultry meat samples. Tang et al. (2020) reported that the *ermB* antimicrobial resistance‐encoding gene was detected in 66.7% of *C. jejuni* and 39.6% of *C. coli* bacteria. They also found that the *tet(O)* gene was detected in all tetracycline‐resistant *Campylobacter* spp. Hull et al. (2021) showed that the majority of *C. jejuni* and *C. coli* bacteria isolated from poultry processing, food animals and retail meat in the United States harboured *tet(O)*, *aadE1*, *aph*, *cmeB* and *blaOXA* resistance genes. A Chinese survey (Du et al., 2018) reported that *Campylobacter* spp. isolated from poultry meat samples carried *tet(O)* (98%), *aadE* (58.90%), *ermB* (20.50%) and *aadE‐sat4‐aphA* (6.60%) antimicrobial resistance‐encoding genes. A similar report was done by Gharbi et al. (2018). They showed that the distribution of *cmeB*, *tet(O)*, *blaOXA‐61* and *aphA‐3* resistance genes among the *C. jejuni* and *C. coli* strains isolated from broiler chickens in Tunisia were 80% and 100%, 100% and 80%, 81% and 93% and 0% and 0%, respectively. Some of the antibiotic‐resistant strains in our survey did not harbour related antimicrobial resistance encoding gene. This part of our survey is in agreement with those of Gharbi et al. (2018) and Marotta et al. (2019). These strains might harbour other genetic determinants conferring antimicrobial resistance. As we could detect both resistance to tetracycline and *tet(O)* gene, resistance to ciprofloxacin and fluoroquinolones and *cmeB* gene, resistance to β‐lactams (ampicillin and amoxicillin/clavulanic acid) and *blaOXA‐61* gene, resistance to aminoglycosides and *aphA‐3* and *aadE1* genes, they would not be a good alternative for the campylobacteriosis treatment. Additionally, as the majority of *Campylobacter* strains harboured *cmeB*, *tet(O)*, *blaOXA‐61* and *aadE1* genes, they might have a major function in mediating antimicrobial resistance against their specific classes of antimicrobials.

The virulence genes involved in motility (*flaA)*, adhesion (*cadF*, *dnaJ* and *racR*), invasion (*pldA*, *virB11* and *ciaB*), cytotoxin production (*cdtA*, *cdtB* and *cdtC*), lipoprotein encoding (ceuE) and GB syndrome (*wlaN* and *cgtB*) were the main genes detected in the *Campylobacter* spp. isolated from the examined poultry meat samples. As a result, consuming raw or uncooked poultry meat can lead to campylobacteriosis and subsequent severe complications. Thus, research on the *Campylobacter* virulence characteristics in food animals, particularly poultry meat, is essential for consumer safety. Rendering to our findings, *flaA* and *ciaB* were detected in all *C. jejuni* and *C. coli* isolates. Additionally, *racR*, *dnaJ*, *cdtB*, *cdtC* and *cadF* were detected in more than 50% of strains. In a similar survey, Fani et al. (2019) reported that all *Campylobacter* isolates were positive for *cdtC*, *cdtB*, *cdtA* and *cadF* virulence factors and the total distribution of *pldA* and *cgtB* were 65.40% and 15.40%, respectively. Gharbi et al. (2018) showed a significant relation between virulence characteristics and antimicrobial resistance of *Campylobacter* strains isolated from poultry meat samples. They reported that ampicillin‐resistant strains harboured racR and ciaB virulence factors, amoxicillin/clavulanic acid‐resistant ones harboured racR, cadF and ciaB, nalidixic acid‐resistant ones harboured racR, and chloramphenicol‐resistant ones harboured cadF and ceuE. However, this relationship between virulence factors and antimicrobial resistance was not determined in the present investigation, but some research indicated an in vitro increased invasion of resistant strains as compared to susceptible ones (Ghunaim et al., 2015). In keeping with this, some other researchers defined the tendency of susceptible strains to cause more severe infections than resistant ones (Feodoroff et al., 2009). Thus, additional studies should be conducted to explore more in‐depth the relationship between the pathogenic traits and the antimicrobial resistance in *Campylobacter* strains. The high distribution of virulence factors was also reported in surveys conducted in the United States (Poudel et al., 2022), Poland (Wieczorek et al., 2018), Brazil (Takeuchi et al., 2022), Pakistan (Melo et al., 2013) and China (Zhang et al., 2016). We found a higher distribution of virulence factors among the *C. jejuni* isolates than *C. coli* bacteria. This finding may show that *C. jejuni* is much more common as a cause of human infections. This interpretation was supported by Melo et al. (2013) and Samad et al. (2019).

In the final section of the present survey, ERIC‐PCR was used for molecular typing of *Campylobacter* spp. according to findings; the majority of isolates had more than 80% genetic similarities and were classified in the same group. This finding may show their common source and route of transmission into the chicken meat samples. Additionally, high diversity was determined between *C. jejuni* and *C. coli* isolated from different raw poultry meat samples. This part of our findings was akin to those reported from South Africa (Igwaran & Okoh, 2020a), Egypt (Ahmed et al., 2015) and India (Milton et al., 2015).

## 5 CONCLUSION

In conclusion, virulent and antimicrobial‐resistant strains of *C. jejuni* and *C. coli* were isolated from chicken, ostrich, turkey, quebec and goose meat samples. Chicken and Turkey meat samples harboured the highest contamination rates. *C. jejuni* and *C. coli* isolates harboured a high resistance rate against erythromycin, ampicillin, ciprofloxacin, chloramphenicol and tetracycline, which was accompanied by the high distribution of *aadE1*, *blaOXA‐61* and *tet(O)* antimicrobial resistance‐encoding genes. These findings may show a change in the pattern of antibiotic resistance of *Campylobacter* isolates compared to previous studies and the need to find alternative antibiotics in the coming years. *FlaA*, *ciaB*, *racR*, *dnaJ*, *cdtB*, *cdtC* and *cadF* were found in the majority of isolates, which shows their high pathogenicity. Isolates that were classified in similar ERIC‐PCR‐based groups may have similar routes of transmission. The role of raw goose and quebec meat samples in the transmission of virulent, and antimicrobial‐resistant *C. jejuni* and *C. coli* to the human community was also determined. Further investigations should perform to compare the antibiotic resistance pattern, virulence gene profile and ERIC‐PCR typing of *C. jejuni* and *C. coli* strains isolated from poultry meat and human being. Additionally, there is a large demand to assess the relationship between virulence characters and antimicrobial resistance properties in *C. jejuni* and *C. coli* isolates.

## AUTHOR CONTRIBUTIONS

HM and AS carried out the molecular genetic studies, participated in the primers sequence alignment and drafted the manuscript. MH and AS carried out the sampling and culture method. HM and AS participated in the design of the study, performed the statistical analysis and writing the manuscript. All authors read and approved the final manuscript.

## CONFLICT OF INTEREST

The authors declare that they have no competing interests.

### ETHICS APPROVAL AND CONSENT TO PARTICIPATE

The research was extracted from the PhD thesis in the field of microbiology and was ethically approved by the Council of Research of the Faculty of Basic Science, Shahrekord Branch, Islamic Azad University, Shahrekord, Iran (Consent Ref Number IR.IAU.SHK.REC.1400.042). Verification of this research project and the licenses related to sampling process were approved by the Prof. Hassan Momtaz (Approval Ref Number MIC201946).

### PEER REVIEW

The peer review history for this article is available at https://publons.com/publon/10.1002/vms3.944.

## Data Availability

All data analysed during this study are included in this published article.
